# Thirty-five year mortality following receipt of SV40- contaminated polio vaccine during the neonatal period

**DOI:** 10.1054/bjoc.2001.2065

**Published:** 2001-09-01

**Authors:** C Carroll-Pankhurst, E A Engels, H D Strickler, J J Goedert, J Wagner, E A Mortimer Jr

**Affiliations:** Mandel School of Applied Social Sciences, Case Western Reserve University, Cleveland, OH, 44106-7164, USA; Viral Epidemiology Branch, National Cancer Institute, Rockville, MD, 20852, USA; Department of Epidemiology and Biostatistics, Case Western Reserve University, Cleveland, OH, 44106-4945, USA

**Keywords:** SV40, poliovirus vaccines, cancer, newborns

## Abstract

Early poliovirus vaccines, both inactivated and live attenuated, were inadvertently contaminated with simian virus 40 (SV40), a monkey virus known to be oncogenic for newborn hamsters. Although large epidemiologic studies have not identified an elevated cancer risk in persons who received SV40-contaminated vaccines, fragments of SV40 DNA have recently been identified in certain human tumours. We report the follow-up of a cohort of 1073 persons, unique because they received SV40-contaminated poliovirus vaccines as newborns in 1961–63. A previous report of the status of these subjects as of 1977–79 identified 15 deaths, none due to cancer. The present study utilized the National Death Index to identify deaths in the cohort for the years 1979–96. Expected deaths were calculated from Cleveland area sex-, age-, race- and year-specific mortality rates. Increased mortality from all causes was not found. 4 deaths from cancer were found compared to 3.16 expected (*P*= 0.77). However, 2 deaths from testicular cancer occurred, compared to 0.05 expected (*P*= 0.002), which may be a chance finding due to multiple comparisons. There were 2 deaths due to leukaemia, a non-significant finding, and no deaths due to tumours of the types putatively associated with SV40. Although these results are, for the most part, consistent with other negative epidemiologic investigations of risks from SV40-contaminated vaccines, further study of testicular cancer may be warranted, and it will be important to continue monitoring this cohort which is now reaching middle-age. © 2001 Cancer Research Campaign


British Journal of Cancer (2001) **85**(9), 1295–1297
© 2001 Cancer Research Campaign
doi: 10.1054/ bjoc.2001.2065, available online at http://www.idealibrary.com on IDEAL

http://www.bjcancer.com

### Thirty-five year mortality following receipt of SV40-contaminated polio vaccine during the neonatal period

**C Carroll-Pankhurst[1], EA Engels[2], HD Strickler[2], JJ Goedert[2], J Wagner[3] and EA Mortimer Jr[3]**

[1]Mandel School of Applied Social Sciences, Case Western Reserve University, Cleveland, OH 44106-7164, USA; [2]Viral Epidemiology Branch, National Cancer Institute, Rockville, MD 20852, USA; [3]Department of Epidemiology and Biostatistics, Case Western Reserve University, Cleveland, OH 44106-4945, USA

**Summary** Early poliovirus vaccines, both inactivated and live attenuated, were inadvertently contaminated with simian virus 40 (SV40), a monkey virus known to be oncogenic for newborn hamsters. Although large epidemiologic studies have not identified an elevated cancer risk in persons who received SV40-contaminated vaccines, fragments of SV40 DNA have recently been identified in certain human tumours. We report the follow-up of a cohort of 1073 persons, unique because they received SV40-contaminated poliovirus vaccines as newborns in 1961–63. A previous report of the status of these subjects as of 1977–79 identified 15 deaths, none due to cancer. The present study utilized the National Death Index to identify deaths in the cohort for the years 1979–96. Expected deaths were calculated from Cleveland area sex-, age-, race- and year-specific mortality rates. Increased mortality from all causes was not found. 4 deaths from cancer were found compared to 3.16 expected ($P = 0.77$). However, 2 deaths from testicular cancer occurred, compared to 0.05 expected ($P = 0.002$), which may be a chance finding due to multiple comparisons. There were 2 deaths due to leukaemia, a non-significant finding, and no deaths due to tumours of the types putatively associated with SV40. Although these results are, for the most part, consistent with other negative epidemiologic investigations of risks from SV40-contaminated vaccines, further study of testicular cancer may be warranted, and it will be important to continue monitoring this cohort which is now reaching middle-age. © 2001 Cancer Research Campaign

**Keywords**: SV40; poliovirus vaccines; cancer; newborns

Between 1959 and 1963 100 million or more Americans received inactivated or live attenuated poliovirus vaccines inadvertently contaminated with simian virus 40 (SV40) (Sweet and Hilleman, 1960; Shah and Nathanson, 1976). SV40 was present in monkey kidney tissue used for propagation of poliovirus. SV40 is oncogenic in newborn hamsters following parenteral inoculation. SV40 serologic studies of human recipients and attempts to recover virus from those given live vaccine yielded suggestive but inconclusive results (Sweet and Hilleman, 1960; Gerber et al, 1961; Shah and Nathanson, 1976).

Subsequent population-based studies in the United States, Germany and Sweden have not provided evidence of excess cancer incidence in persons exposed to SV40 in poliovirus vaccines (Geissler, 1990; Olin and Geisecke, 1998; Strickler and Goedert, 1998; Strickler et al, 1998). However, reports have appeared of SV40 DNA fragments found by PCR in certain uncommon human tumours, such as ependymomas, other brain neoplasms, osteosarcomas in children, and mesotheliomas in adults (Butel and Lednicky, 1999). To some extent this spectrum of tumours parallels that occurring in SV40-infected animals (Stenton, 1998). Uncertainties in, and alternative explanations for, these observations prevent firm conclusions (Geissler, 1990; Carbone et al, 1994; Strickler et al, 1996; Shah et al, 1997; Butel and Lednicky, 1999).

This study is a follow-up of 1073 individuals who received poliovirus vaccines at one to three days of age in 1960 or 1962 in Cuyahoga County, Ohio, to try to determine their immunogenicity in the neonatal period (Lepow et al, 1962). These subjects are unique because the vaccines were shown to contain live SV40 and, like the hamsters who developed SV40-related tumours, they were exposed to SV40 at an age of presumed immunologic immaturity. Previous follow-ups (Fraumeni et al, 1970; Mortimer et al, 1981) uncovered no deaths due to cancer. The recent identification of SV40 DNA fragments in some tumours from other studies has reawakened interest in this cohort.

*Received 17 April 2001*
*Revised 2 July 2001*
*Accepted 24 July 2001*

*Correspondence to:* C Carroll-Pankhurst

### SUBJECTS AND METHODS

To identify deaths of study subjects subsequent to the 1981 report (Mortimer et al, 1981) we used the National Death Index (NDI) which is maintained by the National Center for Health Statistics (NCHS). The NDI was established in 1979 to provide an accurate national method for ascertaining deaths of individuals participating in medical, epidemiologic and other long-term health investigations of large mobile populations (Patterson and Bilgrad, 1986). Data regarding each death are provided to the NDI by the states electronically from the death certificates, and include 14 identifying items, although all 14 are not always available. The NDI search protocol initially comprises a comparison of the researcher's data with the NDI database on 7 specific items to develop a list of potential matches. These potential matches are then subjected to further analyses using the remaining 7 criteria to ascertain their accuracy. The researcher is provided a listing of any matches in descending order of probability including the state in which the death certificate is located. For what appear to be certain matches the NDI also provides the underlying cause of death.

Whether to pursue the death certificates of less certain matches is decided by the researcher. Contact with providers or family members is usually not permitted without consent of the state of death.

15 of the original study group of 1073 infants were known to be deceased by 1979. Information on subjects collected from birth through 1979, including addresses, telephone numbers, and names of parents, siblings and in some instances other family members and contacts, was available. The names and identifying data for the 1058 study subjects believed to be alive in 1979 were submitted to the NDI. The study was approved by the human subjects review boards of the two hospitals where the subjects were born, and by the jurisdictions from which copies of the death certificates were requested when required.

We compared observed and expected numbers of deaths, for various causes, for the period from 1969 (the first year systematic data on non-cancer deaths were available from NCHS) to 1996 (the most recent year for which NDI data were available). Observed deaths were derived from prior follow-up studies (Fraumeni et al, 1970; Mortimer et al, 1981) for 1969–78 and from the NDI search for 1979–96. The expected numbers of deaths from various causes, 1969–96, were determined using NCHS sex-, age-, race- and year-specific mortality rates for Cuyahoga County, Ohio, the area from which the subjects were drawn. Sex or race data were not available for 18 and 797 subjects, respectively. To calculate expected rates, therefore, we imputed fractional values of sex (50% male) and race (83% black, 17% white based on the composition of the remaining cohort. In sensitivity analyses in which we varied these fractions over a range of reasonable values (40–60% for male sex, 70–90% for black race), we found the expected rates for cancer outcomes changed little (data not shown).

We estimated expected numbers of deaths for all cancers combined and for those specific cancers that actually occurred in deceased cohort members. Additionally, we estimated the expected numbers of non-cancer deaths, total and cause-specific for observed non-cancer deaths. Relative risks were calculated for each cause of death (observed deaths divided by expected deaths) (Hennekens and Buring, 1987). Exact confidence intervals and two-sided *P* values for these relative risks were determined using the Poisson distribution.

### RESULTS

The search of the NDI data produced 577 potential matches with study subjects. As ranked by NCHS, the vast majority of these were unlikely to be true matches. Two study personnel independently reviewed each potential match and eliminated 432 for whom the date of birth differed by more than one year and the state of birth was not Ohio. To ensure that no possible matches were missed, death certificates were requested for the remaining 145 subjects even though some of the data items did not match. These included records that had a phonetic match on one or more names, or had one mismatch in the date of birth (either month, or day, or +/– 1 year). Each death certificate was then compared to the original subject records. Agreement on the parents' names and the date and location of birth as well as subject name was necessary to validate each match.

A total of 44 deaths in study subjects were identified (Table 1). Of these, 41 death certificates were obtained through the information provided by the NDI, one was found as a result of a pilot study to assess the feasibility of locating living subjects 20 years after the last contact, and one case occurred in 1977 and was reported previously (Mortimer et al, 1981). The death certificate of a 44th person with an identical, though common, given name, surname, middle initial, birth date, and residence in Ohio at birth and at death according to the NDI could not be located by the state's Division of Vital Statistics, and search of the medical records of the two study hospitals was non-productive. We and the NDI considered this death to be a probable match and it is included in Table 1.

Table 1 shows the causes of death of these 44 persons. There were 3 deaths from cancer confirmed by death certificates. Two were due to testicular tumours and one to myelogenous leukaemia. A fourth cancer death (the probable match noted above) was a case of lymphatic leukaemia. Although there were only two cases of testicular cancer, the relative risk was 37.9 ($P = 0.002$). Also, the relative risk of leukaemia was 4.2 with two cases but this was not significant ($P = 0.16$). Overall, the relative risk of cancer death (all types combined) was 1.27. All 4 subjects who died of cancer had received live, attenuated vaccine as newborns.

We identified no death from brain tumours, osteosarcomas or mesotheliomas. For all cancers other than testicular cancer and

**Table 1** Observed and expected causes of death in 44 persons, 1969 to 1996, who received SV40-contaminated poliovirus vaccine as newborns in 1960 or 1962

| Cause of death | ICD9 Nos. | Observed | Expected | RR | 95% CL |
|---|---|---|---|---|---|
| All | | 44 | 47.15 | 0.93 | 0.67–1.25 |
| All cancer | 140–208, 235–239 | 4 | 3.16 | 1.26 | 0.34–3.23 |
| Testis | 186 | 2 | 0.05 | 36.98 | 4.47–133.50 |
| Leukaemia | 204–208 | 2 | 0.48 | 4.19 | 0.51–15.73 |
| Other cancer | Residual | 0 | 2.62 | – | – |
| Non-cancer | | 40 | 44.00 | 0.90 | 0.64–1.23 |
| External | E800–E999 | 28 | 26.91 | 1.04 | 0.69–1.50 |
| Homicide | E960–E978 | 16 | 13.53 | 1.18 | 0.67–1.91 |
| Suicide | E950–E959 | 5 | 3.75 | 1.33 | 0.43–3.10 |
| Trauma | E800–E949 | 7 | 7.42 | 0.94 | 0.38–1.94 |
| Automobile | E810–E825 | 6 | 4.32 | 1.39 | 0.51–3.03 |
| Other trauma | E800–E809, E826–E949 | 1 | 3.10 | 0.32 | 0.01–1.78 |
| AIDS | 024–044 | 4 | 3.84 | 1.04 | 0.28–2.66 |
| Other non-cancer | Residual | 8 | 13.24 | 0.60 | 0.26–1.18 |

ICD9 numbers used for calculation of expected deaths, RR = relative risk, 95% CL = 95% confidence limits.

*© 2001 Cancer Research Campaign*

leukaemia, the risk actually appeared decreased (no observed deaths vs. 2.62 expected deaths).

Most of the non-cancer deaths were due to violence and other extrinsic causes and rates were similar to those expected in these age, sex and ethnic groups, suggesting that the lack of an overall increase in cancer mortality in the SV40-exposed cohort was not due to under-ascertainment of deaths.

### DISCUSSION

After more than 35 years of follow-up, we found no overall excess of deaths from cancer in these individuals who as infants received poliovirus vaccines contaminated with SV40. The results are reassuring, consonant with other epidemiological studies (Geissler, 1990; Olin and Geisecke, 1998; Strickler and Goedert, 1998; Strickler et al, 1998), and of special interest because the vaccines were given in the very early newborn period, the age of highest risk for oncogenesis induced by SV40 in the hamster model. Notably absent were deaths from tumours induced by SV40 in laboratory animals, including brain tumours, osteosarcomas and mesotheliomas.

Of some interest are the 2 deaths from testicular cancer. One died at 21 years, and the other at 31 years. Testicular tumours have not been observed in animals infected with SV40 and there is no report of SV40 fragments in human testicular tumours. However, SV40 antigens have been described in normal human sperm fluids (Martini et al, 1996). Although it is conceivable that SV40 infection caused testicular cancer in these subjects, at least 2 other explanations are more likely. One obvious explanation is that the apparent excess is due to chance related to multiple comparisons. There were in effect many implied comparisons in this study in that a search of the NDI was made for deaths due to any and all causes of death, the vast majority of which did not appear. It is also relevant that whereas expected values can, as here, be small fractions, observed numbers have to be whole numbers. Another possibility is that, due to their predominately low socioeconomic status, our subjects were at higher risk for mortality from this usually curable tumour because of delayed recognition and treatment; but on the other hand testicular cancer exhibits higher rates in higher socioeconomic groups. Nonetheless, further study of this association in humans is needed.

We found a relative risk for death due to leukaemia of 4.2. SV40 has been shown to induce leukaemia and lymphomas in hamsters (Diamandopoulos, 1972). Interestingly, 2 previous studies reported detection of SV40 in human haematological malignancies (Martini et al, 1998; Rizzo et al, 1999). The significance of these results is unclear (Rizzo et al, 1999), since positive results have also been observed in blood specimens from healthy controls (Martini et al, 1996; Martini et al, 1998) and additional studies confirming such an association have not, to our knowledge, been reported. The 2 leukaemia cases differed in cell types (one myelogenous and one lymphatic). The association between SV40 and leukaemia did not achieve statistical significance and, like the association with testicular cancer, there may be explanations other than causality.

There are several limitations to these results. First, the 35 to 37 year duration of follow-up may be inadequate for some neoplasms implicated in the pathologic studies, such as mesotheliomas, although the central nervous system tumours with identified SV40 fragments are primarily those of childhood and would be expected to have a much shorter latent period. Secondly, the relatively small number of subjects compromises a study of site-specific lesions. Finally, it is possible that some deceased subjects were not identified by the NDI. This would particularly involve females whose names changed due to marriage. However, mortality from non-cancer causes was not low among females, which would have been the case if they were under-ascertained (data not shown).

Future studies of this cohort may prove informative. For example, it may be possible to utilize cancer registry data for the Cleveland area to ascertain cancer incidence, which could provide complementary data to the present study. Also, because the age of the subjects at the time of this follow-up was 35 to 37 years, considerably younger than the ages at which some neoplasms usually occur, the records of this cohort are being retained for possible future use if warranted by other laboratory and epidemiologic information.

### REFERENCES

Butel JS and Lednicky JA (1999) Review. Cell and molecular biology of Simian virus 40: implications for human infections and disease. *J Natl Cancer Inst* **91**: 119–134

Carbone M, Pass HI, Rizzo P, Marinetti MR, DiMuzio M, Mew DJP, Levine AS and Procopio A (1994) Simian virus 40-like DNA sequences in human pleural mesothelioma. *Oncogene* **9**: 1781–1790

Diamandopoulos GTh (1972) Leukemia, lymphoma, and osteosarcoma induced in the Syrian golden hamster by Simian virus 40. *Science* **176**: 173–175

Fraumeni JF, Stark CR, Gold E and Lepow ML (1970) Simian virus 40 in polio vaccine: follow-up of newborn recipients. *Science* **167**: 59–60

Geissler E (1990) SV40 and human brain tumors. *Prog Med Virol* **37**: 211–222

Gerber P, Hottle GA and Grubbs RE (1961) Inactivation of vacuolating virus (SV40) by formaldehyde. *Proc Soc Exp Biol Med* **108**: 205–209

Hennekens CH and Buring JE (1987) *Epidemiology in Medicine*. Little Brown: Boston.

Lepow ML, Warren RJ, Ingram VG, Daugherty SC and Robbins FC (1962) Sabin type 1 oral poliomyelitis vaccine: effect of dose upon response of newborn infants. *Am J Dis Child* **104**: 67–71

Martini F, Iaccheri L, Lazzarin L, Carinci P, Corallini A, Gerosa M, Iuzzolino P, Barbanti-Brodano G and Tognon M (1996) SV40 region and large T antigen in human brain tumors, peripheral blood cells, and sperm fluids from healthy individuals. *Cancer Research* **56**: 4820–4825

Martini F, Dolcetti R, Gloghini A, Iacherri L, Carbone A, Boiocchi M and Tognon M (1998) Simian-virus-40 footprints in human lymphoproliferative disorders of HIV– and HIV+ patients. *Int J Cancer* **78**: 669–674

Mortimer EA, Jr., Lepow ML, Gold E, Robbins FC, Burton GJ and Fraumeni JF, Jr. (1981) Longterm follow-up of persons inadvertently inoculated with SV40 as neonates. *N Engl J Med* **305**: 1517–1518

Olin P and Geisecke J (1998) Potential exposure to SV40 in polio vaccines used in Sweden during 1957: no impact on cancer incidence rates 1960 to 1993. In Brown F, Lewis AM (eds): Simian virus 40 (SV40): A possible human polyomavirus. *Dev Biol Stand* **94**: 227–233

Patterson BH and Bilgrad R (1986) Use of the National Death Index in cancer studies. *J Natl Cancer Inst* **77**: 877–881

Rizzo P, Carbone M, Fisher SG, Matker C, Swinnen LV, Powers A, DiResta I, Alkan S, Pass HI and Fisher RI (1999) Simian virus 40 is present in most United States human mesotheliomas, but it is rarely present in non-Hodgkin's lymphoma. *Chest* **116**: 470S–473S

Shah K and Nathanson N (1976) Human exposure to SV40: review and comment. *Am J Epidemiol* **103**: 1–12

Shah KV, Daniel RW, Strickler HD and Goedert JJ (1997) Investigation of human urine for genomic sequences of the primate polyomaviruses simian virus 40, BK virus, and JC virus. *J Infect Dis* 1618–1621

Stenton SC (1998) Editorial. Simian virus 40 and human malignancy. *BMJ* **316**: 877

Strickler HD and Goedert JJ (1998) Exposure to SV40-contaminated poliovirus vaccine and the risk of cancer-A review of the epidemiological evidence. *Dev Biol Stand* **94**: 235–244

Strickler HD, Goedert JJ, Fleming M, Travis WD, Williams AE, Rabkin CS, Daniel RW and Shah KV (1996) Simian virus 40 and pleural mesothelioma in humans. *Cancer Epidemiol Biomarkers Prev* **5**: 473–475

Strickler HD, Rosenberg PS, Devesa SS, Hertel J, Fraumeni JF, Jr., and Goedert JJ (1998) Contamination of poliovirus vaccines with simian virus 40 (1955–1963) and subsequent cancer rates. *JAMA* **279**: 292–295

Sweet BH and Hilleman MR (1960) The vacuolating virus, SV40. *Proc Soc Exp Biol Med* **105**: 420–427

© 2001 Cancer Research Campaign
